# Cancer stem cells CD133 and CD24 in colorectal cancers in Northern Iran

**Published:** 2016

**Authors:** Anahita Nosrati, Farshad Naghshvar, Iradj Maleki, Fatemeh Salehi

**Affiliations:** 1 *Department of Pathology,* Gastrointestinal Cancer Research Center, *Imam Hospital, Mazandaran University of Medical Sciences, Sari, Iran*; 2 *Department of Pathology, Imam Hospital, Mazandaran University of Medical Sciences, Sari, Iran*; 3 *Gut and Liver Research Center, Department of Gastroenterology, Imam Hospital, Mazandaran University of Medical Sciences, Sari, Iran *

**Keywords:** Cancer stem cells, Colorectal cancer, CD24, CD133, Clinicopathologic characteristics

## Abstract

**Aim:**

We aimed to study the expression of CD24 and CD133 in colorectal cancer and normal adjacent tissues to assess a relationship between these markers and clinic-pathological characteristics and patient’s survival.

**Background:**

Cancer stem cells are a group of tumor cells that have regeneration and multi-order differentiation capabilities.

**Patients and methods:**

Expression of CD24 and CD133 was studied in a paraffin block of colorectal cancer and normal tissues near tumors with the immuneohistochemical method in patients who were referred to Imam Khomeini Hospital in Sari.

**Results:**

A total of 50 samples (25 males and 25 females) with a mean age of 67.57±13.9 years old with range 28-93 years, included 3 mucinous carcinoma and 47 adenocarcinoma. Expression of CD133 marker was negative in 29 cases and positive in 21 cases. Expression of CD24 in tissue near tumor cells was found in 30% of available samples. The relationship between expressing CD24 with treatment (surgery and chemotherapy) was significant and its relationship with patient’s survival was insignificant statistically. However, there was a clear difference as mean survival age of patients based on CD24 expression was 26.64±18.15 for negative cases and 41.75±28.76 months for positive cases. CD24 and CD133 expressions and their co-expression with other clinic-pathological factors were not significant.

**Conclusion:**

During this study, the relationship between CD24 and treatment type was significant. To confirm this result, various studies with high sample numbers and other stem cell markers are recommended.

## Introduction

 Colorectal cancer is a second to third common cancer and one of the major leading causes of cancer death in both females and males in the West. Colorectal cancer is responsible for more than 500,000 deaths annually throughout the world (1,2). In Iran, colorectal cancer is the third most common cancer in men and fourth common cancer in women (except skin cancer), and its outbreak is increasing (5000 new cases in a year) (1, 2). 

In recent years, a concept named cancer stem cells (CSCs) has been prevalent. CSCs, which have stem cell characteristics (i.e. abilities of self-renewal (self-repair) and differentiation) was first expressed in 1997 for blood malignancies and solid tumor of colon cancer (3-5).

Identifying these cells in colon cancer “colorectal cancer stem cell (CO-CSC)” was conducted with expression of some cell surface markers in various studies (3-6). Cancer stem cells were effective in trigger, completion, metastasis and recurrence of tumors, as well as resistance against the ordinary selective treatment of colon cancer (e.g. chemotherapy) and recurrence of tumor(7). CO-CSC markers are identified as pre-warning factors in colon cancer survival and there are data in scientific papers about developing treatments as CSC target (8).

CD24 is a cell surface marker with mono-chain sialoglycoprotein and 24 KD molecular weights expressed in many solid tumors such as cancer stem cells in pancreas cancer(9). CD24 is a small protein consisted of 27 amino-acids, which were glycosylated with certain method and linked to the cell membrane via phosophatidyl inositol playing role in transferring extracellular messages inside of cells. CD24 is expressed in 50.5% of colon cancer patients. The relationship between this marker and clinic-pathological factors (10), as well as metastasis to lymph node (11) and shorter survival was reported(12) .

CD133 or prominin-1 gene codes, which is a membrane glycoprotein on chromosome 4p15.32, has a role in connecting stem cells to their sites, maintaining polarity, as well as movement and cell-cell or cell-matrix interferences. The relationship between expressions of this marker and clinico-pathologic characteristics of patients were reported, including gender, microscopic grade stage, depth of invasion survival, resistance to chemotherapy, and involvement of lymph node (11-13).

The 5-year survival rate for colorectal cancer patients is 50% in dependent from treatment(9) which in the case of metastasis reaches to 10% and death of most patients is because of tumor metastatic expansion to other organs(10) .Because of high outbreak of colorectal cancer in north of Iran and increasing trend of this cancer in world and in young people(14) this study was conducted to identify cancer stem cells in colorectal cancers using immunohistochemically method(IHC) and to determine the association between expression of CD24 and CD133 with clinicopathologic parameters and survival of colorectal cancer patients. 

## Patients and Methods

***Patients and samples***

In this study, paraffin blocks of 50 patients with colorectal cancer were selected. Patients had referred to Imam Khomeini hospital in Sari during 2001-2012. Verification and completion of clinico-pathologic data were done using hematoxylin and eosin (H&E) stained pathologic slides, pathology record, hospital file and contact with patients. Clinico-pathologic parameters were age, gender, location, type of tumor, depth of invasion, lymph node metastasis and distant metastasis (including liver), tumor differentiation, vascular, lymphatic invasion, treatment, recurrence, survival (time in a month) and current condition (alive/dead because of the tumor or its side effects). Patients who had chemotherapy or family polypus history or IBD before surgery were excluded. All samples were fixed in neutral buffered formalin 10% and paraffin blocked. All cut sections from both tumor and adjacent normal colorectal mucosa were selected and stained immunohistochemically.

***Immunohistochemistry staining procedure***

Tissue cut sections with 3-4 micrometer thickness were prepared and stained by H&E. After eliminating paraffin by immersing in Xylene, watering with descending ethanol concentration (99%, 95% and 77%), tissues were placed in buffer TRIS-EDTA (pH=9) and transferred to microwave with a maximum power reaching to melting point. After microwave power was reduced to 40%, samples were kept for 15 minutes and were placed in room temperature for 15 minutes. After washing with tap water and TBS buffer, DAKO pen was used to determine the periphery (extent) of tissue and placed in a wet room and incubated using detection kit for mouse monoclonal antibody (Anti human CD24 100 µg and Anti CD133 100 µg) with 1/100 dilution for one night in room temperature. Then, samples were covered by peroxides for 30 minutes, and chromomeric reaction was conducted with a relevant kit. Positive control kits for CD24 and CD133 were tonsil lymph tissue. Our negative control was the tissue that was covered with just buffer. Samples were placed in room temperature and hematoxylin was used as background stain. 

**Figure 1:**
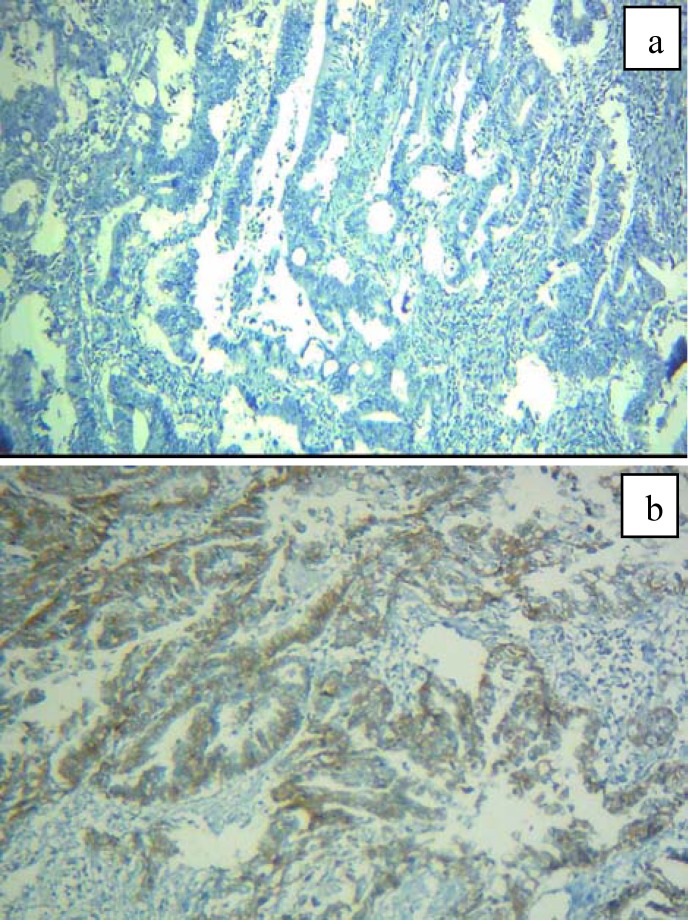
Colorectal adenocarcinoma. a and b, immunohistochemically negative and positive CD24 expression 400X

**Figure 2:**
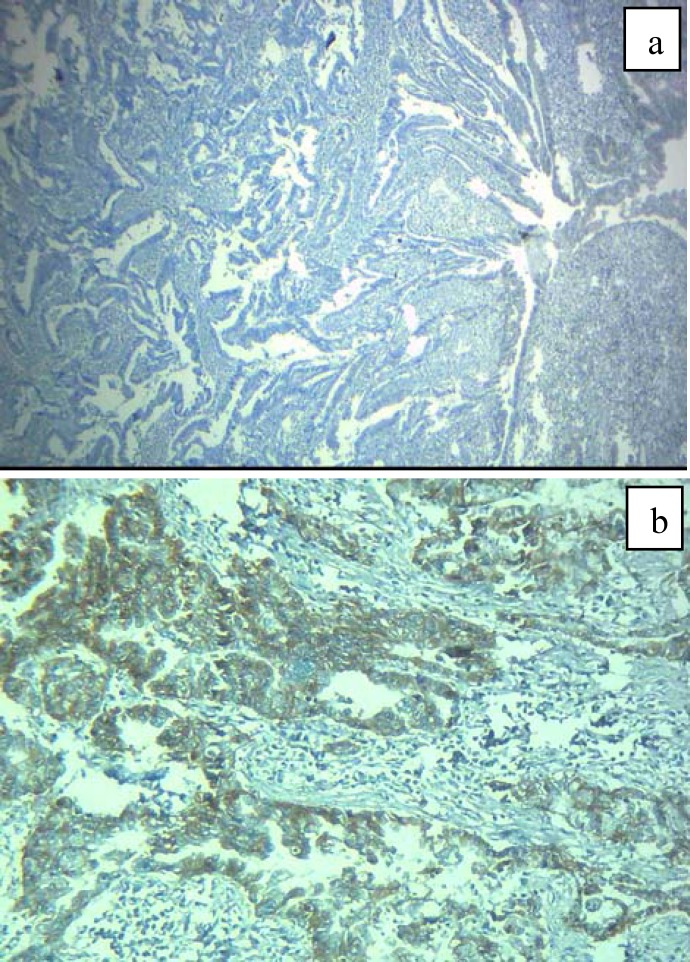
Colorectal adenocarcinoma. a and b, negative and positive CD133 cytoplasmic expression, Immunohistochemistry 400x

Prepared specimens were studied semi-quantitatively by two expert pathologists using light microscope (Labomed LX400) in low and high powers (100X, 400X). All cytoplasmic and membranous staining were observed and scored. In the case of CD24 marker, staining was less and more than 5% of tumor cells considered negative and positive, respectively (11,12) (Figure 1a and b). For CD133, stained tumor cells less and more than 50% were determined as negative and positive respectively (13) (Figure 2a and b).

***Statistical analysis***

Patients’ data and immunohistochemistry results were performed by SPSS software (IBM SPSS statistics 20.0.1) using X^2^, Fisher exact test, student and paired T-test. P-value below 0.05 was significant. 

## Results

In 50 patients, mean age was 64.57±13.9 years with range 28-93 years old. Twenty-five patients were male. The mean age of men and woman was 65.71±12.11 and 63.48±15.62 years, respectively. There was no significant difference between two groups (p=0.58). Tumor type in 47 subjects was adenocarcinoma (prevalence in male and female was 88% and 100%, respectively) and in 3 subjects were mucinous carcinoma (just in men). However, there was no significant relationship between gender and tumor pathology (p >0.05). It is worth mentioning that the mean age of patients suffering from adenocarcinoma and mucinous carcinoma were 65.37±13.8 and 52.33±10.7 years old, respectively. No significant difference was identified between two groups.

CD24 expression in 29 cases was negative. Mean age of patients with negative marker was 63.24±12 and in patients with positive marker was a 63.24±16.3 year, which was not statistically significant. Based on pathologic findings, expression of CD133 was positive in 45 patients. In 19 cases (38%), both markers were positive. Markers expression has been summarized in Chart-1. Mean age of negative CD133 patients was 65.5±14.1 years and there was no significant difference between both groups (p=0.94). Mean age of patients with positive and negative expressions was 63.4±17.1 and 65.3±11.7 years old, and no significant difference was evident between two groups. Frequency distribution of gender, based on CD24 in negative group was 40% and in positive group was 51.1%, and no significant difference was observed between both groups. Frequency distribution of gender had no statistically significant difference based on the expression of two markers expression.

Regarding the type of tumor (adenocarcinoma /mucinous carcinoma), there was no significant relationship between tumor nature and expression of CD24 and CD133. The mean size of tumors in all studied patients was 4.57±1.94 cm with a 1-10cm range. The relationship between tumor sizes and the expression of these two markers was not significant. Most tumors were in right colon (54%) and rectum (38%). Thirty-one cases (62%) were well differentiated, 14 cases (28%) were moderately differentiated and 3 cases were poorly differentiated. CD24 and CD133 co-expression based on tumor differentiation was not significantly different. Regarding CD24, 17 cases in negative and 16 cases in positive groups (58.2% versus 76.2%) and regarding CD133, 4 cases of negative and 29 cases of positive groups were well differentiated, and showed no significant difference. Interestingly, in co-expressed and non-co-expressed groups, differentiation was not significantly different. No significant relationship between these markers and depth of invasion was observed. In addition, their co-expression showed no significant relationship. Among 50 patients, 20 patients (40%) had lymph node metastasis, and in this regard lymph node metastasis showed no significant difference between the markers and their co-expression as well (P>0.05). 

Based on results, 31(62%), 12(24%) and 5 (10%) patients were in M0, M1A and MIB stages (the condition of two cases was unknown), respectively. According to data, frequency of distant metastasis based on both co-expression and expression of CD24 and CD133 alone had no significant difference. Among 50 studied patients, 11 patients (22) had liver metastasis, which was based on CD24 and CD133 expression alone or co-expression no significant difference was seen. 

In evaluating results regarding disease stage, 5 (10%), 16(32%), 4(8%), 3(6%), and 13(26%) patients were in stage I, IIA, IIB, IIIA, and IIIB stage, respectively (9 patients had not known stage). Regarding the markers, the disease stage had not a significant difference. In 10 patients (20%), blood vessel invasion was observed and in 27 patients (54%). Regarding CD24, blood vessel invasion was not significant. This held for CD133 and co-expression of CD24 and CD133. In 7 patients (14%), lymphatic vessel invasion was seen (condition of 13 patients was unknown), but no significant difference based on co-expression of two markers was observed.

According to the treatment method, 9(18%), 30(60%), 3(84%) patients treated with surgery, surgery and chemotherapy, as well as surgery along with other methods, respectively. Eight patients (16%) underwent surgery accompanied by other unknown treatments. Interestingly, the relationship between CD24 marker with treatment was seen in cases who underwent both surgery and chemotherapy (P=0.04). However, this relationship was not obtained in the CD133 positive cases and co-expression of both markers. The mean survival time for patients from the time of diagnosis was 33.83±24.66 months with a range of 1-120 months. Although there was no statistically significant difference between both groups, this 14-month-time difference in survival is considerable. Recurrence was found in 10(20%) patients after primary treatment (it was not known in 11 patients). Recurrence occurred in 28.6% and 22.2% of negative and positive CD24 patients, respectively. However, the difference between two groups was not significant. Regarding the CD133, recurrence was seen in 20% and 26.5% negative and positive patients and no significant difference was observed. Recurrence took place in 3 co-expressed and 7 non-co-expressed patients, which was insignificant in the analysis. During the study, 20 patients (40%) were died due to colorectal cancer and 21 patients (42%) were alive (condition of 9 patients was not available). No significant difference based on marker expression was obtained. Summary of results regarding the relation between clinicopathologic criteria and expression of markers was shown in table 1. 

**Table 1:** CD24 and CD133 expression in colorectal cancers and their relation with clinicopathologic characteristics

Tumor Characteristics		CD24 Negative (%)	CD24 Positive (%)	p-value	CD133 Negative (%)	CD133 Positive (%)	p-value
Tumor site	Right colon Left colon Rectum	19(5.65) 1(4.3) 9(31)	8(40) 2(10) 10(50)	0.18	30(60) 0(0) 2(40)	24(5,54) 3(8.6) 17(6.38)	0.99
Tumor Differentiation	Well Moderately Poorly	17(58.6) 9(31) 3(3.10)	16(76.2) 5(8.23) 0(0)	0.55	4(80) 1(20) 0(0)	29(64.4) 13(28.9) 3(7.6)	0.99
Depth of Invasion	T2 T3 T4	1(2.4) 10(7.41) 13(2.4)	3(20) 6(40) 6(40)	0.33	1(50) 0(0) 1(50)	3(1.8) 16(2.43) 18(6.48)	0.2
Lymph node Metastasis	No Yes	15(7.51) 14(3.48)	15(4.71) 6(6.28)	0.16	1(20) 4(80)	19(2.42) 26(8.57)	0.64
Distant Metastasis	M0 MIA MIB	18(7.66) 6(22.2) 3(1.11)	13(9.61) 6(6.28) 2(5.9)	0.9	3(60) 2(40) 0(0)	28(1.65) 10(21.3) 5(6.11)	0.63
Stage	I IIA IIB IIIA IIIB	1(2.4) 8(3.33) 3(5.12) 3(5.12) 9(5.37)	4(5.23) 8(1.47) 1(9.5) 0(0) 4(5.23)	0.18	0(0) 1(50) 0(0) 0(0)	5(8.12) 15(5.38) 4(3.10) 3(7.7) 12(8.30)	0.99
Liver Metastasis	No Yes	22(5.81) 5(5.18)	15(4.71) 6(6.28)	0.15	4(80) 1(20)	33(7.76) 10(3.23)	0.99

**Figure 3:**
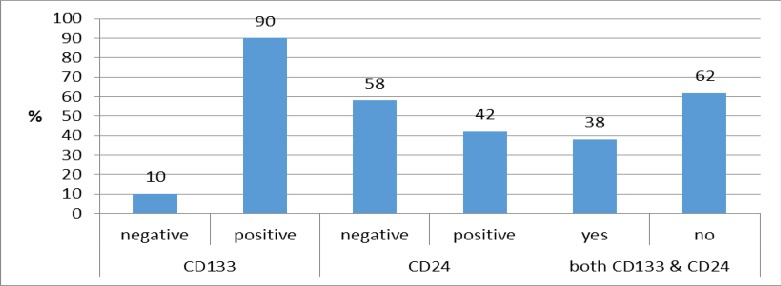
Colorectal adenocarcinoma. a and b, negative and positive CD133 cytoplasmic expression, Immunohistochemistry 400x

## Discussion

Cancer stem cell (CSC) has been used as a new concept in scientific articles since 1997 (3, 5-7). The relationship of these cells with the solid tumor, such as breast, prostate, brain, lung cancer, neuroblastoma, rhabdomyosarcoma and renal cell carcinomas, as well as intestine cancers were studied (3, 5-15). It is important to study CD24 and CD133 markers, due to their relationship with metastasis and recurrence, as well as their resistance to anti-cancer treatments like chemotherapy (5).

Few studies showed increased expressions of CD24 and CD133 as a primary event in colorectal carcinogens (16-18). Up to now, there has not been a common cut off for CD133 and various studies used various methods. However, we found the cut off %50 is rather practical regarding Labarge et al. study. Choi D et al. studied the expression of CD24 and CD133 with IHC method on colorectal adenocarcinoma and showed an association between the expression of CD24 and CD133 with tumor differentiation, as well as the relation of CD133 with male gender and depth of tumor invasion (9) these findings were not found in our study. Wang et al. stated that CD24 has an indirect effect on tumor cell proliferation and tumor growth (19). The relationship between CD133 in colon cancer with male gender (13) and lymph node involvement was reported as well (12, 15). Some studies have presented the relationship of this marker with depth of tumor invasion (13, 15) and tumor grade (11); however, some studies have rejected it (7).

In animal studies, Baumann used the flow cytometry method in cancer cells of rat and reported the influence of CD24 in tumor growth (tumor size) and metastasis (20). Iinuma showed the expression of CK/CD133 had prognostic effect on Duke’s stage B and C (20, 21). On the other hand, Lim and Weichert and Ahmed et al. did not show a significant relationship between CD24 and clinico-pathological factors like age, gender, tumor type or differentiation (16, 22). This finding is in agreement with our study. It seems that this can stem from the limited number of studies on these new markers, and low number of available appropriate colorectal specimens. Su and Lim et al. displayed that CD24 expression is accompanied by lymph node metastasis in colon cancer (17). This result was not seen in our study and Dangho et al. study (9,16).

 Regarding the role of CD24 in intercellular junctions, it is claimed that its expression may enhance metastasis (23,24). Moreover, other researchers showed the relation of CD133 and CD 24 co-expression in colon cancer with liver metastasis (25-27). In comparison with the latter, such relation was not found in our and the other studies (9) which can be referred to different characteristics of tumor cells in metastases.

In their studies, Weichert (16) and Lee supported the relationship between CD24 and low survival (28). However, Choi (9) and Ahmed et al. (22) did not explain this relationship. Although it was not statistically significant in our study, we encountered about 14- month difference between the positive and negative groups. Thus, as far as the survival is concerned, this difference can be effective in making a decision in the follow up and treatment of patients. Regarding CD133, Horst et al. showed that 5- and 10-year survival in high CD133 was less than low CD133 cases (26). Few studies also supported CD133 role in predicting patients’ survival (29). Our study showed no relationship between CD133 expression and patients’ survival, which is in agreement with other studies (9). The worst prognosis in CD133 positive cases in the former study was due to the higher tumor grade in their specimens. Kijima et al. showed that both negative and positive CD133 tumors had no difference in survival time (30). There were also other studies, which showed poor survival in CD133 positive colorectal tumor. However, these studies were conducted on limited patients after chemotherapy (29, 31). According to Gazaniga et al. study, there was no relationship between CD133 expression on tumor cells and survival of patients with colorectal cancer (32). Chemotherapy and radiotherapy resistance are essential problems, which influence survival of colorectal patients (33).

In this study, we found positive (cytoplasmic and membrane) expression of CD24 related to treatment (both surgery and chemotherapy), and somehow survival although it was not significant statistically. On the other hand, there was no relationship between CD133 expressions and clinico-pathologic factors in colorectal cancer. Furthermore, co-expression of these two markers had no significant effect on tumor characteristics. This indicates by evaluating the expressions of these markers on larger samples of colorectal cancer, we can help pre-awareness of patients suffering from colorectal cancers and come up with valuable information. This will also help to improve anti-stem cells treatment strategies.

## Note

**(**Please cite as: **Nosrati** **A,**** Naghshvar** **F, Maleki I,** **Salehi F. Cancer stem cells CD133 and CD24 in colorectal cancers in Northern Iran. Gastroenterol Hepatol ****Bed Bench**** 2016;9(2):132-139).**
